# Chloroplast genome sequence of the moss *Tortula ruralis*: gene content, polymorphism, and structural arrangement relative to other green plant chloroplast genomes

**DOI:** 10.1186/1471-2164-11-143

**Published:** 2010-02-27

**Authors:** Melvin J Oliver, Andrew G Murdock, Brent D Mishler, Jennifer V Kuehl, Jeffrey L Boore, Dina F Mandoli, Karin DE Everett, Paul G Wolf, Aaron M Duffy, Kenneth G Karol

**Affiliations:** 1 USDA-ARS-MWA, Plant Genetics Research Unit, University of Missouri, 205 Curtis Hall, Columbia MO, 65211, USA; 2 Department of Integrative Biology and University and Jepson Herbaria, 1001 Valley Life Sciences Bldg, University of California, Berkeley, Berkeley, CA 94720-2465, USA; 3 Physical Biosciences, Lawrence Berkeley National Laboratory, 1 Cyclotron Rd, Berkeley, CA, 94720 USA; 4 Genome Project Solutions, Inc, 1024 Promenade St, Hercules, CA 94547, USA; 5 Department of Biology, University of Washington, 106 Kincaid, Seattle, WA, 98195, USA; 6 Department of Biology, Utah State University, 5305 Old Main Hill, Logan, UT, 84322, USA; 7 The Lewis B. and Dorothy Cullman Program for Molecular Systematics Studies, 200th St & Southern Boulevard, The New York Botanical Garden, Bronx NY 10458, USA

## Abstract

**Background:**

*Tortula ruralis*, a widely distributed species in the moss family Pottiaceae, is increasingly used as a model organism for the study of desiccation tolerance and mechanisms of cellular repair. In this paper, we present the chloroplast genome sequence of *T. ruralis*, only the second published chloroplast genome for a moss, and the first for a vegetatively desiccation-tolerant plant.

**Results:**

The *Tortula *chloroplast genome is ~123,500 bp, and differs in a number of ways from that of *Physcomitrella patens*, the first published moss chloroplast genome. For example, *Tortula *lacks the ~71 kb inversion found in the large single copy region of the *Physcomitrella *genome and other members of the *Funariales*. Also, the *Tortula *chloroplast genome lacks *petN*, a gene found in all known land plant plastid genomes. In addition, an unusual case of nucleotide polymorphism was discovered.

**Conclusions:**

Although the chloroplast genome of *Tortula ruralis *differs from that of the only other sequenced moss, *Physcomitrella patens*, we have yet to determine the biological significance of the differences. The polymorphisms we have uncovered in the sequencing of the genome offer a rare possibility (for mosses) of the generation of DNA markers for fine-level phylogenetic studies, or to investigate individual variation within populations.

## Background

*Tortula ruralis *(Hedw.) Gaertn., also known as *Syntrichia ruralis *(Hedw.) F. Weber & D. Mohr (Pottiaceae) is a moss with a cosmopolitan distribution in relatively dry habitats. In North America the species is widespread in northern latitudes but is more common in the Western U.S., south into Mexico [1]. *Tortula ruralis *has received considerable attention over the last forty years as a model for the study of vegetative desiccation tolerance, i.e., the ability to equilibrate to the water potential of dry air and survive, regaining growth and development upon rehydration. *Tortula ruralis *offers much as an experimental model for the study of environmental impacts on plants: it grows easily in culture, has a limited number of cell types, and, because of its morphology, experimental treatments act directly at the cellular level [2,3]. It is the latter property that also makes it an ideal choice for an indicator species in air pollution studies [4,5].

*Tortula ruralis *is among the most desiccation-tolerant of land plants and it can recover from desiccation even after at least three years in the dried state [6,7]. *Physcomitrella patens *is relatively tolerant of dehydration but cannot tolerate the levels of drying that *T. ruralis *can survive [8]. It is well established that the chloroplast plays a central role in the recovery of vegetative plants cells from desiccation [9] and it is possible that differences between the chloroplast genomes of *T. ruralis *and *P. patens *may relate to this fundamental difference between the two mosses.

The rapid recovery of photosynthesis is critical in order to recover and re-establish growth when water is available, thus maximizing the time available to the moss for carbon fixation and productivity [10]. Following slow drying to -100 Mpa, photosystem II (PSII) activity in *T. ruralis *recovers within minutes after rewetting [11], with normal rates of carbon fixation returning within an hour [2]. Photosynthesis is essential for the production of the energy required for repair and protein synthesis following the desiccation event. Obviously, the integrity and metabolic capacity of the chloroplast is central to the speed of recovery of photosynthesis. It is clear from electron microscopic observation of freeze-fracture preparations that chloroplast membranes, both the envelope and thylakoid membranes, in *T. ruralis *are unaltered by desiccation [12], which supports the idea that desiccation does not damage the photosynthetic apparatus. Such protection of chloroplast structure has also been demonstrated for gametophytes of *Polytrichum formosum*, which also appear to be unaltered by the imposition of desiccation and the rigors of rehydration [9]. Thus it is clear that the chloroplast holds a central role in the response of *T. ruralis *to desiccation and rehydration and it is important to study the nature of its genome in this plant, the first vegetatively desiccation-tolerant plant to have its chloroplast genome sequenced. The genome sequence of *T. ruralis*, and its comparison to other chloroplast genomes, is critical if we want to understand how the interplay between the nuclear and chloroplast genomes plays a role in desiccation tolerance.

In addition to the relevance of the *T. ruralis *chloroplast genome to the important trait of desiccation tolerance, the genome sequence has considerable relevance to our current understanding of evolutionary history of the land plants. Current evidence suggests that mosses are the sister group of hornworts plus tracheophytes, diverging at least 450 million years ago [13,14]. As an early diverging lineage, mosses hold a place in the phylogeny of land plants that is important for comparative purposes to seed plants [15], although comparisons are currently hampered because only one published chloroplast genome is available for mosses, while hundreds are available for its sister group. Several chloroplast genome sequences will be required to estimate the ancestral genome sequence for mosses, which will in turn allow comparisons with tracheophyte genomes.

The interest in *Tortula ruralis *as a model desiccation-tolerant organism has increased as our need to understand how plants survive dehydration stress grows and the global impact of climate change becomes more critical. This impetus and the need for increasing sampling within the mosses for phylogenetic comparative purposes led to the choice of *T. ruralis *for chloroplast genome sequencing. The assembly and annotation of the *T. ruralis *chloroplast genome sequence is presented here, only the second chloroplast genome sequenced for a moss and the first for a desiccation tolerant plant. The first chloroplast genome for a moss, *Physcomitrella patens*, was completed in 2003 [16]. Because *P. patens *was found to have a major rearrangement in the chloroplast compared to what PCR-based methods show for most other moss lineages [17], the *T. ruralis *chloroplast genome will serve as an important point of comparison and will assist in ongoing efforts to utilize whole-genomic sequences and structural characters in a comparative phylogenetic framework [13,18].

## Results

### Whole chloroplast genome description

**Figure 1:**
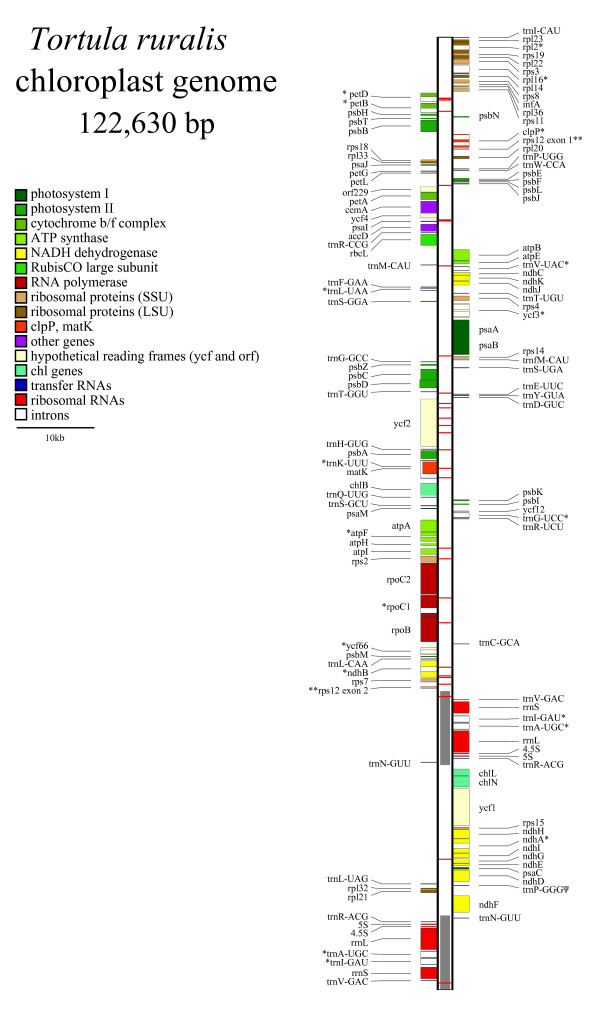
***Tortula ruralis *chloroplast genome structure**. Genes (colored boxes) on the right side of the map are transcribed in the top down direction, whereas those on the left side are transcribed bottom up. The tRNA genes are indicated by the one-letter amino acid code (fM = initiator methionine) followed by the anticodon. Introns are show with an asterisk (*), the trans-spliced gene *rps12 *is shown with two asterisks (**) and the pseudogene *trnP*^GGG ^is shown with a Psi (Ψ). Horizontal red lines along the genome indicate polymorphic nucleotides.

**Figure 2:**
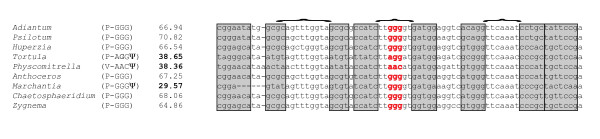
**Alignment of *trnP*^GGG ^coding regions from *Tortula *and eight other green plant chloroplast genomes**. Columns from left to right are: Species name, tRNA anticodon, and Cove score for tRNA structure derived from tRNAscan-SE [23]. The stem regions of the tRNA molecule are indicated by grey boxes with corresponding internal stem segments indicated by brackets: the tRNA anticodon region is shown in red. Alteration of the anticodon region is found in both moss lineages *Tortula ruralis *and *Physcomitrella patens*.

The chloroplast genome sequence comprises 122,530 bp; a gap of ~750 bp (an estimate based on comparison to *Physcomitrella patens*) remains undetermined within the coding region of *ycf2 *despite repeated attempts to sequence this region using long-distance PCR and gene walking. While the precise length of the chloroplast genome remains unknown, it is estimated to be 123.5 kb. Figure 1 summarizes the structure of the *T. ruralis *chloroplast genome and additional file 1 lists the genes contained within it and their relative nucleotide positions. Structurally, *T. ruralis *lacks the large ~71 kb inversion in the LSC region of the genome that is found in *P. patens*. The gene list reveals the absence of *petN*, a gene that is found in all other known land plant chloroplast genomes. The *trnP*^GGG ^gene, although containing an altered anticodon region similar to that seen in *Physcomitrella*, also contains significant mismatches in the stem regions of the predicted tRNA structure. The inference is that this gene has become a pseudo gene in both lineages (Figure 2.). The gene content of the inverted repeat (IR) regions is conserved between *T. ruralis *and *P. patens*.

### Polymorphisms

One surprising discovery was the identification of apparent polymorphism in the *T. ruralis *chloroplast DNA. Overall, we observed 29 clearly polymorphic sites, each of which appears to have two possible states (Table 1). Two of the polymorphisms appear in the non-coding region of the IR and so only 28 of the polymorphic sites are unique. Eleven of the sites were situated within protein coding sequences: five result in synonymous amino acid codons and six are non-synonymous. The remaining polymorphic sites are situated in non-coding regions: six in introns and ten in the intergenic regions.

**Table 1:** List of all sequence polymorphisms located on the *Tortula ruralis *chloroplast genome sequence.

position	location	state	strand	codon
7810	**petD intron**	Y (C/T)	-	
7821	**petD intron**	M (A/C)	-	
8076	**petD intron**	M (A/C)	-	
9525	**petB intron**	Y (C/T)	-	
19020	**psbJ/petA IGR**	K (G/T)	na	
23480	**psaI/ycf4 IGR**	K (G/T)	na	
23623	**psaI/ycf4 IGR**	K (G/T)	na	
29394	**trnM/trnV IGR**	M (A/C)	na	
41006	**psaB/rps14 IGR**	R (A/G)	na	
45811	**trnT/trnE IGR**	W (A/T)	na	
47128	**ycf2**	R (A/G)	-	GTA or GTG - synonymous
47698	**ycf2**	M (A/C)	-	TTA or TTC - nonsynonymous
49026	**ycf2**	K (G/T)	-	GTA or TTA - nonsynonymous
49946	**ycf2**	K (G/T)	-	AGT or ATT - nonsynonymous
50907	**ycf2**	R (A/G)	-	TAA or TAG - synonymous
53115	**trnH/psbA IGR**	R (A/G)	na	
55502	**matK**	K (G/T)	-	TTG or TTT - nonsynonymous
56736	**trnK intron**	Y (C/T)	-	
65765	**atpI/atpH IGR**	K (G/T)	na	
67161	**rps12**	R (A/G)	-	CGA or CGG - synonymous
72206	**rpoC1-exon2**	K (G/T)	-	TGT or TTT - nonsynonymous
75462	**rpoB**	Y (C/T)	-	CGC or CGT - synonymous
81152	**ndhB intron**	R (A/G)	-	
82247	**ndhB exon1**	Y (C/T)	-	CCA or TCA nonsynonymous
82463	**rps7/ndhB IGR**	W (A/T)	na	
83334	**rps12 exon2/rps7 IGR**	W (A/T)	na	
84909	**Noncoding IR**	K (G/T)	na	
105822	**ndhG**	M (A/C)	+	GTA or GTC - synonymous
121800	**Noncoding IR**	M (A/C)	na	

## Discussion

### Whole chloroplast genome description

Compared to other published chloroplast genomes, the chloroplast genome of *Tortula ruralis *is most similar to that of *Physcomitrella patens*. However, *T. ruralis *lacks the large ~71 kb inversion in the LSC region of the genome that is found in *P. patens*. This large inversion is found only in the moss order *Funariales*, whereas other moss lineages have a more plesiomorphic gene order similar to the liverwort *Marchantia polymorpha *[16,17].

It is interesting that the *Tortula ruralis *chloroplast genome lacks the *petN *gene because the protein encoded by this gene plays an important role in photosynthesis electron transport. The PetN protein is a subunit of the cytochrome b6f (Cyt b6f) complex of cyanobacteria and plants. The Cytb6f complex is a plastoquinol-plastocyanin oxidoreductase within thylakoid membranes that functions as the photosynthetic redox control of energy distribution between the two photosystems, PSII and PSI, and gene expression [19]. With such a critical role in chloroplast function it is clear that *T. ruralis *cannot be devoid of the PetN polypeptide and it is very likely, given the conservation of gene order in plant chloroplasts, that the *petN *gene does not reside in the region of the genome for which we do not yet have a sequence (e.g., within *ycf2*). What remains is the probability that in *T. ruralis *the *petN *gene has moved into the nuclear genome or there is a nuclear-encoded gene product that serves the same function as the PetN protein. This hypothesis remains to be tested but it offers an exciting speculation that the translocation of the *petN *gene from the chloroplast to nuclear genome may be related to the stability of the chloroplast in desiccation-tolerant mosses. Obviously, a much more detailed survey of moss chloroplast genomes needs to be accomplished and further experimental evidence needs to be collected before such speculation can be tested.

### Polymorphism

Few studies have detected nucleotide polymorphism for plastid DNA within plant populations [20]. Our results suggest a considerable level of population polymorphism in the *Tortula ruralis *chloroplast DNA. Mosses have monoplastidic cell division [21,22], thus it is unlikely that the polymorphism occurs within individual plants. Because the sequenced material was grown from wild-collected spores from multiple parents, the polymorphism is more likely to be due to genotypic races or otherwise cryptic lineages within the source population. One alternative source of polymorphism that is difficult to discount completely is that the variation is caused by inclusion of nuclear DNA which can occasionally contain fragments of chloroplast DNA [23]. We consider this unlikely because our source of plastid DNA was from isolated chloroplasts, so nuclear DNA levels would be very low and the chances of capturing rare inserts would be negligible. Also, the 29 polymorphisms were distributed across the genome rather than clustered in one region which is what would be expected if one or a few nuclear regions had been captured. Many recently sequenced plastid genomes have used shotgun or PCR-based approaches, and completely eliminating nuclear DNA is impossible.

The polymorphisms that give rise to non-synonymous changes in amino acid at a defined site in a protein coding region are possible targets for post transcriptional editing should the non-synonymous change result in a loss or detrimental change in protein function. For example, the polymorphic position 744 in the *matK *gene (A or C) could result in either leucine (UUA) or phenylalanine (UUC) in the resultant peptide. Given the importance of this group II intron maturase gene to chloroplast function [24] this change either does not result in a significant change in the normal activity of the polypeptide or it would be corrected by the RNA editing activity within the chloroplast prior to translation. Testing this hypothesis, and estimating the extent of RNA editing will require sequencing of cDNA from *Tortula *chloroplast genes.

The shotgun sequencing method used for assembling this genome makes a determination of the number of haplotypes in the sequenced sample impossible. However, there are some indications that only two haplotypes may be present: (1) all variable sites were bimodal (e.g., either an A or G); and (2) in 5 instances, multiple polymorphic sites were located within a single DNA fragment and in all 5 cases only two versions of the sequence were present. Interestingly, one third of the variable sites occurred in coding regions of the chloroplast, including two sites in *rbcL *and three in *ndhF*. Polymorphic sites that can be easily sequenced from chloroplast DNA have potential for future use in population-level studies in *T. ruralis*. Future studies that include cloning and sequencing chloroplasts genomic regions from single individuals, gathered from across the range of this species, would be very productive. It would also be of interest to determine if the polymorphisms within the coding regions of the chloroplast genes generate functional allelic variation in the gene products involved.

### trnP^GGG^

Among the known chloroplast genomes of land plants, the *trnP*^GGG ^gene shows an unusual evolutionary pattern. In *Physcomitrella patens *and *Tortula ruralis *the sequence data indicate that this gene has become a pseudogene in these lineages (Figure 2); further substantiated by the low Cove scores for the tRNA structure prediction from tRNAscan-SE [25]. In the liverwort *Marchantia polymorpha*, the anticodon is intact but there are stem mismatches and a deletion of 5 bases, indicating that this gene may also be pseudogenized in *M. polymorpha*. However, in the hornwort *Anthoceros formosae *and in vascular plants *trnP*^GGG ^appears to be functional. It is of course possible that the change in the anticodon sequence seen in *T. ruralis *and *P. patens *are modified post-transcriptionally to allow non-wobble interactions and thus rendering the tRNA functional. However, if these alterations in the *trnP*^GGG ^seen in *T. ruralis *and *P. patens *do indicate a loss of function then given the current understanding of land plant phylogeny, this would imply at least two independent deactivation events for the *trnP*^GGG ^gene in the chloroplast. Functional copies of *trnP*^GGG ^occur in the chloroplast genomes of the green algae *Chaetosphaeridium *and *Zygnema*, otherwise this gene appears to have been lost in *Chara *and is absent in other examined green-algal chloroplast genomes [26]. The GGG codon is used in coding regions of the chloroplast with roughly equal frequency in all of these lineages.

### Relationships to desiccation-tolerance

Protection of chloroplast structure appears to be a major aspect of the mechanism for desiccation tolerance in bryophytes (for review see Oliver [27]). It is unclear at the moment as to how much the chloroplast genome, and the genes it encodes, influences the stability of the chloroplast during dehydration, or how much it contributes to the rapid resumption of photosynthetic electron transport. However, the importance of the chloroplast in the phenotype of desiccation tolerance cannot be underestimated. This is evident in the percentage of transcripts encoding chloroplast-directed proteins that are present in the transcriptome of recovering rapid-dried gametophytes of *Tortula ruralis *[28]. Of the transcripts that can be annotated in the rehydration transcriptome, 12.5% are classified as chloroplastic in the Gene Ontology (GO) functional classification scheme. One of the more prominent being the transcript that encode the Early Light Inducible Protein (ELIP) which is described as being an important protein for chloroplast structural protection during desiccation and upon rehydration of *T. ruralis *gametophytes [29]. Unfortunately, because of the nature of plastid transcripts (lack of a polyA tail) they are generally not present in the cDNA preparations used for transcriptome sequencing projects and thus we know little of how their synthesis responds to desiccation and rehydration or what is the role of the chloroplast transcriptome in the mechanism of desiccation tolerance we see in *T. ruralis*. Sequencing the chloroplast genome is an important first step in answering these questions about the interplay between the two genomes, nuclear and plastid, in this important trait. The role of the chloroplast genome and the expression of its genes remains a fertile area for study.

## Conclusions

The *Tortula ruralis *genome differs from that of the only other published chloroplast genome sequence, that of *Physcomitrella patens*. The differences in chloroplast genome structure and gene content offer some tantalizing hypotheses in relation to one of the fundamental differences between the two mosses in their ability to tolerate the stresses associated with extreme water loss. The most intriguing observation is the loss of the *petN *gene, presumably to the nuclear genome, given its important role in photosynthetic electron transport and the significance of this with regards to desiccation tolerance.

The polymorphisms we have uncovered in the sequencing of the genome offer the possibility of the generating chloroplast DNA markers for future fine-level phylogenetic studies, or for future population genetic studies examining individual variation within populations.

## Methods

### DNA Isolation and Sequencing

Material of *Tortula ruralis *for DNA extraction was grown in sterile culture from spores collected from a wild population located under a pine canopy along the southern bank of the Bow River west of Calgary, Alberta, Canada, approximately 51° 06' 04° N, 114° 17' 10° W (voucher specimen deposited in the University and Jepson Herbaria, University of California, Berkeley, CA). Isolated sporophytes with intact capsules were carefully removed from the parent gametophyte, washed twice in 5% bleach solution each followed by a sterile water wash, and placed on sterile minimal media and the spores removed by breaking open the capsules. The moss cultures used in the preparation of chloroplasts for genome sequencing were derived from five different capsules. Isolation of intact chloroplasts was achieved using fluorescence-activated cell sorting (FACS) wherein fluorescently stained organelles can be visualized, separated, and collected using flow cytometry. Total chloroplast DNA was amplified using rolling circle amplification (RCA) with random hexamer primers [30]. Amplified products were sheared in approximately equal-sized fragments of ~3000 bp by repeated passage through a narrow aperture using a Hydroshear (Genemachines, San Carlos, CA). The resulting fragments were subcloned and sequenced by the Department of Energy Joint Genome Institute (JGI). Detailed methods for the complete process can be found in Wolf et al., [31].

### Genome Assembly, Finishing & Annotation

Because the sequences covered random sections of the chloroplast, sufficient numbers of DNA fragments were sequenced to provide an average of eight times coverage for the majority of the genome. Sequence fragments were assembled using the Phrap software package, and Consed was used to visualize assembly of contigs (see http://www.phrap.org). Remaining gaps, low-coverage regions, and areas of questionable assembly were manually resequenced from specific clones or from genomic DNA. The finishing sequences were obtained by cloning, and sequencing PCR-generated fragments obtained using flanking primers from known sequence or by use of a GenomeWalker™ kit (Clontech, Mountain View, CA) using either purified chloroplast genomic DNA or total genomic DNA extractions that contain significant levels of chloroplast DNA.

The resulting genome was annotated using the on-line Dual Organellar GenoMe Annotator (DOGMA) [32] and tRNAscan-SE [25], and gene content was compared to published annotated chloroplast genomes and in particular to the chloroplast genome of *Physcomitrella patens *(NC_005087). Location of introns, pseudogenes, and beginning and endpoints of many genes was aided by the libraries of plastid DNA and annotated genomes available through NCBI GenBank http://www.ncbi.nlm.nih.gov[33].

The inverted repeat (IR) characteristic of the chloroplast genome in embryophytes causes problems for automatic alignment programs such as Phrap, because the two IR copies cannot be differentiated by the program. However, this apparent problem can be turned into an advantage and can be used to determine the ends of the IR. Consed (an assembly viewing program) visually indicates when paired sequences from the same clone are placed too far apart in a contig, making the general area of the IR visually apparent if sufficient reads are presented. A consensus sequence for this region with an additional ~2 kb on either side was excised, and all reads were then aligned to this sequence. Using the "color means match" option in Consed, the ends of the IR were visually determined by finding the points at which there was a transition from all reads agreeing to the presence of two distinct sequence motifs.

The sequence of the chloroplast genome for *Tortula ruralis *(=*Syntrichia ruralis*) was deposited into the NCBI GenBank http://www.ncbi.nlm.nih.gov[33] and given the accession number FJ546412.

## Authors' contributions

MJO cultured and maintained the experimental material, conducted the finishing sequencing, participated in assembly and sequence analysis, and drafted the manuscript, AGM and BDM participated in the genome assembly, data analysis and comparative analyses, JVK and JLB sequenced the isolated chloroplast DNA, ensured quality sequence data, and participated in assembly of the genome, DFM and KDE were involved in the conception of the project, isolated (FACS) and purified the chloroplast DAN for sequencing, PGW, APD and KGK were involved in the conception of the project, participated in the assembly of the genome, annotated the genome, and participated in the comparative analyses. All authors have read and approved the final manuscript.

## Supplementary Material

Additional file 1 **Table S1: *Tortula ruralis *chloroplast genome gene list**. List of all genes annotated for the chloroplast genome of *Tortula ruralis*, indicating protein products position on genome and strand. Exons are listed separately for genes with introns. Click here for file
