# Supplementary material for: Chloroplast genome sequence of the moss Tortula ruralis: gene content, polymorphism, and structural arrangement relative to other green plant chloroplast genomes
Source: BMC Genomics. 2010 Feb 27;11:143. doi: 10.1186/1471-2164-11-143 (PMC2841679; doi:10.1186/1471-2164-11-143)
Supplement: Additional file 1 — Table S1: Tortula ruralis chloroplast genome gene list. List of all genes annotated for the chloroplast genome of Tortula ruralis, indicating protein products position on genome and strand. Exons are listed separately for genes with introns. [file 1471-2164-11-143-S1.DOC]

**Table S1**. **Tortula ruralis chloroplast genome gene list**.

| **Name** | **Protein Product** | **Start** | **End** | **strand** |
| --- | --- | --- | --- | --- |
| **trnI-CAU** | tRNA-Ile | 63 | 136 | + |
| **rpl23** | ribosomal protein L23 | 360 | 635 | + |
| **rpl2 ex1** | ribosomal protein L2 | 665 | 1061 | + |
| **rpl2 ex2** | ribosomal protein L2 | 1670 | 2106 | + |
| **rps19** | ribosomal protein S19 | 2163 | 2441 | + |
| **rpl22** | ribosomal protein L22 | 2459 | 2818 | + |
| **rps3** | ribosomal protein S3 | 2858 | 3514 | + |
| **rpl16 ex1** | ribosomal protein L16 | 3622 | 3630 | + |
| **rpl16 ex2** | ribosomal protein L16 | 4351 | 4782 | + |
| **rpl14** | ribosomal protein L14 | 4901 | 5269 | + |
| **rps8** | ribosomal protein S8 | 5527 | 5925 | + |
| **infA** | translational initiation factor 1 | 6040 | 6276 | + |
| **rpl36** | ribosomal protein L36 | 6384 | 6497 | + |
| **rps11** | ribosomal protein S11 | 6639 | 7031 | + |
| **petD ex1** | cytochrome b6/f complex subunit IV | 8333 | 8325 | - |
| **petD ex2** | cytochrome b6/f complex subunit IV | 7690 | 7214 | - |
| **petB ex1** | cytochrome b6 | 9765 | 9757 | - |
| **petB ex2** | cytochrome b6 | 9170 | 8532 | - |
| **psbH** | photosystem II phosphoprotein | 10140 | 9916 | - |
| **psbN** | photosystem II protein N | 10229 | 10360 | + |
| **psbT** | photosystem II protein T | 10568 | 10461 | - |
| **psbB** | photosystem II CP47 chlorophyll apoprotein | 12237 | 10711 | - |
| **clpP ex1** | clp protease proteolytic subunit | 12540 | 12608 | + |
| **clpP ex2** | clp protease proteolytic subunit | 13246 | 13536 | + |
| **clpP ex3** | clp protease proteolytic subunit | 14019 | 14252 | + |
| **rps12 ex1** | ribosomal protein S12 | 14391 | 14504 | - |
| **rps12 ex2** | ribosomal protein S12 | 83839 | 83585 | - |
| **rpl20** | ribosomal protein L20 | 15345 | 15695 | + |
| **rps18** | ribosomal protein S18 | 16046 | 15819 | - |
| **rpl33** | ribosomal protein L33 | 16265 | 16068 | - |
| **psaJ** | photosystem I subunit IX | 16559 | 16431 | - |
| **trnP-UGG** | tRNA-Pro | 16958 | 17031 | + |
| **trnW-CCA** | tRNA-Trp | 17145 | 17218 | + |
| **petG** | cytochrome b6/f complex subunit V | 17354 | 17241 | - |
| **petL** | cytochrome b6/f complex subunit VI | 17579 | 17484 | - |
| **psbE** | photosystem II cytochrome b559 alpha subunit | 18192 | 18443 | + |
| **psbF** | photosystem II cytochrome b559 beta subunit | 18453 | 18572 | + |
| **psbL** | photosystem II protein L | 18608 | 18724 | + |
| **psbJ** | photosystem II protein J | 18843 | 18965 | + |
| **orf197** | Unknown open reading frame | 19328 | 19921 | - |
| **petA** | cytochrome f | 20952 | 19993 | - |
| **cemA** | chloroplast envelope membrane protein | 22652 | 21147 | - |
| **ycf4** | photosystem I assembly protein ycf4 | 23315 | 22761 | - |
| **psaI** | photosystem I subunit VIII | 23799 | 23689 | - |
| **accD** | accD | 25030 | 24092 | - |
| **trnR-CCG** | tRNA-Arg | 25247 | 25175 | - |
| **rbcL** | rbcL | 26838 | 25411 | - |
| **atpB** | ATP synthase CF1 beta subunit | 27349 | 28827 | + |
| **atpE** | ATP synthase CF1 epsilon subunit | 28834 | 29235 | + |
| **trnM-CAU** | tRNA-Met | 29388 | 29316 | - |
| **trnV-UAC ex1** | tRNA-Val | 29519 | 29555 | + |
| **trnV-UAC ex2** | tRNA-Val | 30085 | 30121 | + |
| **ndhC** | NADH-plastoquinone oxidoreductase subunit 3 | 30304 | 30669 | + |
| **ndhK** | NADH-plastoquinone oxidoreductase subunit K | 30660 | 31409 | + |
| **ndhJ** | NADH-plastoquinone oxidoreductase subunit J | 31448 | 31957 | + |
| **trnF-GAA** | tRNA-Phe | 32229 | 32157 | - |
| **trnL-UAA ex1** | tRNA-Leu | 32657 | 32620 | - |
| **trnL-UAA ex2** | tRNA-Leu | 32348 | 32299 | - |
| **trnT-UGU** | tRNA-Thr | 32961 | 33033 | + |
| **rps4** | ribosomal protein S4 | 33334 | 33942 | + |
| **trnS-GGA** | tRNA-Ser | 34081 | 33997 | - |
| **ycf3 ex1** | hypothetical chloroplast RF34 | 34284 | 34408 | + |
| **ycf3 ex2** | hypothetical chloroplast RF34 | 35122 | 35332 | + |
| **ycf3 ex3** | hypothetical chloroplast RF34 | 36001 | 36153 | + |
| **psaA** | photosystem I P700 apoprotein A1 | 36411 | 38663 | + |
| **psaB** | photosystem I P700 apoprotein A2 | 38689 | 40893 | + |
| **rps14** | ribosomal protein S14 | 41072 | 41374 | + |
| **trnfM-CAU** | tRNA-Met | 41523 | 41594 | + |
| **trnG-GCC** | tRNA-Gly | 41844 | 41774 | - |
| **psbZ** | photosystem II protein Z | 42300 | 42112 | - |
| **trnS-UGA** | tRNA-Ser | 42530 | 42618 | + |
| **psbC** | photosystem II CP43 chlorophyll apoprotein | 44164 | 42743 | - |
| **psbD** | photosystem II protein D2 | 45173 | 44112 | - |
| **trnT-GGU** | tRNA-Thr | 45699 | 45628 | - |
| **trnE-UUC** | tRNA-Glu | 45945 | 46017 | + |
| **trnY-GUA** | tRNA-Tyr | 46113 | 46194 | + |
| **trnD-GUC** | tRNA-Asp | 46383 | 46456 | + |
| **ycf2** | Hypothetical | 52703 | 46582 | - |
| **trnH-GUG** | tRNA-His | 53097 | 53023 | - |
| **psbA** | photosystem II protein D1 | 54282 | 53221 | - |
| **trnK-UUU ex1** | tRNA-Lys | 56809 | 56773 | - |
| **trnK-UUU ex2** | tRNA-Lys | 54510 | 54469 | - |
| **matK** | maturase K | 56245 | 54671 | - |
| **chlB** | protochlorophyllide reductase ChlB subunit | 58978 | 57440 | - |
| **trnQ-UUG** | tRNA-Gln | 59263 | 59192 | - |
| **psbK** | photosystem II protein K | 59540 | 59707 | + |
| **psbI** | photosystem II protein I | 60076 | 60186 | + |
| **trnS-GCU** | tRNA-Ser | 60370 | 60285 | - |
| **psaM** | photosystem I protein M | 60740 | 60642 | - |
| **ycf12** | hypothetical chloroplast RF12 | 60986 | 61087 | + |
| **trnG-UCC ex1** | tRNA-Gly | 61222 | 61244 | + |
| **trnG-UCC ex2** | tRNA-Gly | 61862 | 61899 | + |
| **trnR-UCU** | tRNA-Arg | 61975 | 62046 | + |
| **atpA** | ATP synthase CF1 alpha subunit | 63677 | 62154 | - |
| **atpF ex1** | ATP synthase CF0 subunit I | 64916 | 64361 | - |
| **atpF ex2** | ATP synthase CF0 subunit I | 64142 | 63721 | - |
| **atpH** | ATP synthase CF0 subunit III | 65460 | 65215 | - |
| **atpI** | ATP synthase CF0 subunit IV | 66591 | 65845 | - |
| **rps2** | ribosomal protein S2 | 67502 | 66792 | - |
| **rpoC2** | RNA polymerase beta' subunit | 71735 | 67659 | - |
| **rpoC1 ex1** | RNA polymerase beta | 74578 | 74156 | - |
| **rpoC1 ex2** | RNA polymerase beta | 73452 | 71836 | - |
| **rpoB** | RNA polymerase beta subunit | 77840 | 74601 | - |
| **trnC-GCA** | tRNA-Cys | 78067 | 78137 | + |
| **ycf66 ex1** | hypothetical chloroplast RF66 | 79504 | 79399 | - |
| **ycf66 ex2** | hypothetical chloroplast RF66 | 78868 | 78555 | - |
| **psbM** | photosystem II protein M | 79777 | 79673 | - |
| **trnL-CAA** | tRNA-Leu | 80079 | 79999 | - |
| **ndhB ex1** | NADH-plastoquinone oxidoreductase subunit 2 | 82391 | 81666 | - |
| **ndhB ex2** | NADH-plastoquinone oxidoreductase subunit 2 | 81024 | 80251 | - |
| **rps7** | ribosomal protein S7 | 83020 | 82553 | - |
| **trnV-GAC** | tRNA-Val | 85243 | 85314 | + |
| **16S riRNA** | 16S ribosomal RNA | 85524 | 87019 | + |
| **trnI-GAU ex1** | tRNA-Ile | 87337 | 87373 | + |
| **trnI-GAU ex2** | tRNA-Ile | 88166 | 88201 | + |
| **trnA-UGC ex1** | tRNA-Ala | 88261 | 88298 | + |
| **trnA-UGC ex2** | tRNA-Ala | 89072 | 89106 | + |
| **23S rRNA** | 23S ribosomal RNA | 89259 | 92063 | + |
| **4.5S rRNA** | 4.5S ribosomal RNA | 92175 | 92277 | + |
| **5S rRNA** | 5S ribosomal RNA | 92482 | 92600 | + |
| **trnR-ACG** | tRNA-Arg | 92824 | 92897 | + |
| **trnN-GUU** | tRNA-Asn | 93369 | 93298 | - |
| **chlL** | chlL | 94180 | 95055 | + |
| **chlN** | protochlorophyllide reductase ChlN subunit | 95095 | 96510 | + |
| **ycf1** | hypothetical protein | 96704 | 101485 | + |
| **rps15** | ribosomal protein S15 | 101667 | 101933 | + |
| **ndhH** | NADH-plastoquinone oxidoreductase subunit 7 | 101989 | 103164 | + |
| **ndhA ex1** | NADH-plastoquinone oxidoreductase subunit 1 | 103166 | 103720 | + |
| **ndhA ex2** | NADH-plastoquinone oxidoreductase subunit 1 | 104423 | 105013 | + |
| **ndhI** | NADH-plastoquinone oxidoreductase subunit I | 105083 | 105634 | + |
| **ndhG** | NADH-plastoquinone oxidoreductase subunit 6 | 105739 | 106320 | + |
| **ndhE** | NADH-plastoquinone oxidoreductase subunit 4L | 106361 | 106663 | + |
| **psaC** | photosystem I subunit VII | 106848 | 107093 | + |
| **ndhD** | NADH-plastoquinone oxidoreductase subunit 4 | 107193 | 108692 | + |
| **trnL-UAG** | tRNA-Leu | 109016 | 108939 | - |
| **trnP-GGG** | tRNA-Pro | 109149 | 109222 | + |
| **rpl32** | ribosomal protein L32 | 109687 | 109508 | - |
| **rpl21** | ribosomal protein L21 | 110120 | 109770 | - |
| **ndhF** | NADH-plastoquinone oxidoreductase subunit 5 | 110470 | 112629 | + |
| **trnN-GUU** | tRNA-Asn | 113340 | 113411 | + |
| **trnR-ACG** | tRNA-Arg | 113885 | 113812 | - |
| **5S rRNA** | 5S ribosomal RNA | 114227 | 114109 | - |
| **4.5S rRNA** | 4.5S ribosomal RNA | 114534 | 114432 | - |
| **23S rRNA** | 23S ribosomal RNA | 117439 | 114637 | - |
| **trnA-UGC ex1** | tRNA-Ala | 118448 | 118411 | - |
| **trnA-UGC ex2** | tRNA-Ala | 117637 | 117603 | - |
| **trnI-GAU ex1** | tRNA-Ile | 119371 | 119335 | - |
| **trnI-GAU ex2** | tRNA-Ile | 118542 | 118508 | - |
| **16S rRNA** | 16S ribosomal RNA | 121182 | 119687 | - |
| **trnV-GAC** | tRNA-Val | 121466 | 121395 | - |

List of all genes annotated for the chloroplast genome of Tortula ruralis, indicating protein products position on genome and strand. Exons are listed separately for genes with introns.
